# Liver and muscle hemojuvelin are differently glycosylated

**DOI:** 10.1186/1471-2091-12-52

**Published:** 2011-09-21

**Authors:** Yuzo Fujikura, Jan Krijt, Emanuel Nečas

**Affiliations:** 1 Institute of Pathophysiology and Center of Experimental Haematology First Faculty of Medicine, Charles University in Prague U Nemocnice 5, 12853 Prague 2, Czech Republic

## Abstract

**Background:**

Hemojuvelin (HJV) is one of essential components for expression of hepcidin, a hormone which regulates iron transport. HJV is mainly expressed in muscle and liver, and processing of HJV in both tissues is similar. However, hepcidin is expressed in liver but not in muscle and the role of the muscle HJV is yet to be established. Our preliminary analyses of mouse tissue HJV showed that the apparent molecular masses of HJV peptides are different in liver (50 kDa monomer and 35 and 20 kDa heterodimer fragments) and in muscle (55 kDa monomer and a 34 kDa possible large fragment of heterodimer). One possible explanation is glycosylation which could lead to difference in molecular mass.

**Results:**

We investigated glycosylation of HJV in both liver and muscle tissue from mice. PNGase F treatment revealed that the HJV large fragments of liver and muscle were digested to peptides with similar masses, 30 and 31 kDa, respectively, and the liver 20 kDa small fragment of heterodimer was digested to 16 kDa, while the 50 kDa liver and 55 kDa muscle monomers were reduced to 42 and 48 kDa, respectively. Endo H treatment produced distinct digestion profiles of the large fragment: a small fraction of the 35 kDa peptide was reduced to 33 kDa in liver, while the majority of the 34 kDa peptide was digested to 33 kDa and a very small fraction to 31 kDa in muscle. In addition, liver HJV was found to be neuraminidase-sensitive but its muscle counterpart was neuraminidase-resistant.

**Conclusions:**

Our results indicate that different oligosaccharides are attached to liver and muscle HJV peptides, which may contribute to different functions of HJV in the two tissues.

## Background

Mammalian iron homeostasis is largely regulated by hepcidin. Increased levels of hepcidin result in iron deficiency while decreased expression causes iron overload. Gene mutations affecting the gene HFE2, which encodes HJV protein, result in the absence of hepcidin and cause juvenile hemochromatosis [1]. The importance of HJV as a key component of hepcidin expression has been further underlined by studies which implicate HJV in interactions with BMPs, matriptase-2, and neogenin in crucial mechanisms of induction of hepcidin transcription in liver [2-5]. Expression and processing of HJV protein have been investigated extensively [6-13]. However, many features of HJV still remain unexplained. For example, the roles of muscle HJV are still unknown. Several HJV peptides have been detected by immunoblotting but the definitive mechanisms of HJV processing and details of modification such as glycosylation have not been fully elucidated. In addition, despite its importance, direct studies of HJV from tissues are rare.

HJV is identical to repulsive guidance molecule C (RGMc), a member of the RGM family. It shares key structural characteristics with RGMa and RGMb: an N-terminal signal peptide for targeting to the endoplasmic reticulum (ER), a potential integrin-binding RGD tri-amino acid motif, a partial von Willebrand factor type D domain in which an auto-cleavage site is located, and a C-terminal GPI-anchoring domain [14]. Also two of the three N-glycosylation sites of mouse RGMc/HJV align with those of RGM family members. In addition, all RGMs are known to interact with neogenin [15] and are required for BMP signaling [16]. However, RGMc appears to differ from the others in expression and function. RGMa and RGMb are expressed in the developing and adult central nervous system [17,18], and a known function of mouse RGMa is neural tube closure [19]. RGMc/HJV is mainly expressed in the liver, skeletal muscle, and heart and has never been detected in the nervous system [17]. So far its key role in hepcidin expression in liver is its sole known function. Previously, two membrane-anchored HJV (m-HJV), a 50-55 kDa monomer and a heterodimer consisting of 20 and 35 kDa peptides, and soluble HJV (s-HJV) around 30-50 kDa have been detected in cultured cells, tissues (liver, skeletal muscle, and heart), blood, and serum [2,6,7,9,10,20,21]. Recently, three distinct transcripts of RGMc/HJV in muscle that vary in the length of the 5' untranslated region have been identified [22].

Glycosylation is a common post-translational modification. Glycoproteins participate in important biological processes such as receptor activation, signal transduction, and endocytosis [23]. N-glycosylation takes place at asparagine residues of Asn-X-Ser/Thr motifs when core glycan consisting of 14 sugars is co-translationally attached to newly synthesized polypeptides in the lumen of the ER: the attached glycans play a pivotal role in protein folding, oligomerization, quality control, sorting, and transport. They are subsequently subjected to extensive modification in the Golgi complex, where substrate molecules are progressively modified by the full panel of modifying enzymes to produce a wide diversity of structure [24,25]. In O-glycosylation, oligosaccharides are attached to serine and threonine residues; O-linked glycans may serve for cell signaling, prevention of protein phosphorylation, or regulation of protein turnover [26]. Structural variations in the glycan repertoire at the cell surface produce numerous biomarkers, some of which correlate with differentiation, cell activation, and disease [23]; this suggests that differently glycosylated proteins can have different roles.

We studied HJV from mouse tissues because HJV is located on the extracellular surface/space of the cell membrane where it can interact with molecules on the same cell as well as those of neighboring cells in any direction. The surrounding environment of HJV in tissues may not be duplicated in cell culture systems. Therefore, we examined HJV in liver and muscle, comparing samples from HJV wild type (WT) and knock out (KO) mice, with two N-glycosidases, PNGase F and Endo H, and also with O-glycosidase together with neuraminidase, an exoglycosidase. The detected differences may provide insights into possible different roles of HJV in the two tissues.

## Methods

### Extraction and PI-PLC treatment

Hemojuvelin WT and KO mice (deletion of exon 2 of the Hfe2 gene) were a generous gift from Prof. Silvia Arber, Basel, Switzerland [27]. All animal experiments were approved by the Ethics Committee of the First Faculty of Medicine, Charles University in Prague. Tissue protein was extracted with TBS (20 mM Tris pH 7.4 with 150 mM NaCl) containing 10 mM EDTA, 1% Triton X-100, and a protease inhibitor cocktail (Roche). Extracts were obtained by homogenizing 100 mg of tissue with 500 μL of the buffer at 4°C and subsequent centrifugation at 16,000 × g for 20 minutes at 4°C. Protein concentration was measured by Bradford reagent (Sigma). Phosphatidylinositol-specific phospholipase C (PI-PLC) treatment to release GPI anchor-protein complex from the membrane was done by incubating 100 μL of the extract (around 5 mg of protein) with 150 mU of PI-PLC (Sigma) and a protease inhibitor cocktail in 150 μL of 60 mM Tris-HCl pH 7.4 at 37°C for 3 hours. The reaction mixture was centrifuged at 16,000 × g for 30 minutes at 4°C and the clear supernatant was separated from the membrane precipitate.

### Immunoaffinity purification

Immunoaffinity beads were prepared according to the manufacturer's instruction. At first, six μg of anti-mouse RGM-C antibody (R &D Systems) was incubated with 25 μL suspension of magnetic beads coated with protein G (Dynabeads Protein G, Invitrogen) and then the bound IgG was cross-linked to the beads by Bis (sulfo succinimidyl) suberate (BS^3^) (Sigma). PI-PLC treated samples which contain 400 μg of protein in around 20 μL were incubated with the immunoaffinity beads for 30 minutes with tilting and rotating at room temperature. After washing with TBS containing 0.1% Tween 20 (TBS/T) immunoaffinity-purified proteins were eluted from the beads by 15 μL of 100 mM Glycine-HCl pH 2.8 after 3 minutes of incubation and the eluates were immediately neutralized with 1 μL of 1 M Tris-HCl pH 8.8.

### Glycosylation Study

Glycosidase treatment was carried out based on the protocols of New England BioLabs. Typically, PI-PLC treated samples containing 60 μg of protein were combined with the denaturing buffer containing SDS and DTT in 12 μl reaction mixture and then were heated at 100°C for 10 minutes. Subsequently, corresponding reaction buffer, NP-40 if necessary, and glycosidase(s) were added. The final reaction mixtures, 16 to 20 μL, were incubated at 37°C for one to two hours. Peptide N-glycosidase F was obtained from Sigma (P7367), Endoglycosidase H (P0702), endo-a-N-acetylgalactosaminidase (P0733), and neuraminidase (P0720) were form New England BioLabs.

### Immunoblotting

Proteins were separated on 10% SDS-PAGE under reducing or non-reducing condition and transferred to PVDF membrane (GE Healthcare) by electroblotting. The standard procedure using TBS/T for washing, 5% (w/v) non-fat milk in TBS/T for blocking, 16 hours incubation at 4°C with the primary antibody (anti-mouse RGM-C, R and D systems; 0.3 μg/mL), and 2 hours incubation at room temperature with the peroxidase-conjugated anti-goat secondary antibody (Jackson Immuno Research; 1:1000) was employed. Positive signals were visualized on X-ray film by chemiluminescence using detection reagents (Cell Signaling Technology). For the loading control the same membrane was treated, after washing with stripping buffer (ABcam protocol), with anti-GAPDH antibody (Sigma, G9545; 2.5 ng/mL) and subsequently with anti-rabbit secondary antibody (Jackson Immuno Research; 1:1000). Mark 12 Unstained Standard (Invitrogen, LC5677) and Sigma Marker wide Range (Sigma, S8445) were used to estimate the molecular mass of protein bands.

## Results

Detection of minor proteins in extract from animal tissues by immunoblotting encounters difficulties because of non-specific positive signals due to high contents of certain other proteins and a high background. Therefore, an optimal condition for the immunodetection of HJV using a commercial antibody was established by analyzing extracts from WT and KO mice in reducing and non-reducing SDS-PAGE. The use of KO extracts enabled us to determine non-specific positive signals and eliminate them from consideration. Based on previous reports and comparison of WT and KO samples, we determined HJV peptides in tissue. Figures are representatives of more than two experiments. The molecular masses are approximations based on two commercial standards.

### Detection of tissue hemojuvelin

**Figure 1:**
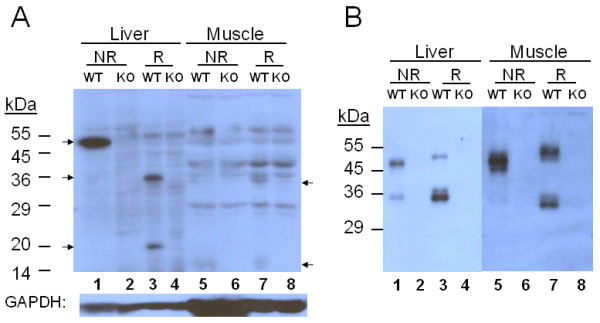
**HJV in Liver and Muscle**. **A**. Extracts containing 60 μg of protein from HJV WT and KO mice were analyzed by 10% SDS-PAGE under non-reducing (NR) and reducing (R) conditions. Arrows indicate HJV peptides. **B**. Immunoaffinity-purified samples were analyzed by 10% SDS-PAGE under non-reducing (NR) and reducing (R) conditions. HJV peptides were detected by immunoblotting.

In liver samples (Figure 1A, 1-4), under non-reducing conditions a thick positive band which contains single chain monomers and heterodimers was clearly detected around 50 kDa and under reducing conditions distinctive 35 and 20 kDa bands, two fragments of the heterodimer, were detected in WT but not in KO samples. In muscle samples (Figure 1A, 5-8), many non-specific bands appeared in both WT and KO samples and no clear positive bands were detected under non-reducing condition. However, a positive band at 34 kDa, possibly the large fragment of the heterodimer, was detected uniquely in WT but not in KO samples under reducing conditions. Interestingly, the muscle small fragment which is expected to be around 20 kDa was not detected. Instead, a smudge was detected at 14-18 kDa in WT but not in KO samples under both conditions. In both liver and muscle samples, 50-55 kDa monomers were not clearly detected even under reducing conditions at this stage.

### Immunoaffinity Purification

There may have been HJV peptides whose quantities in the extracts were very low, and thus went undetected in the previous blot. Therefore, in order to see a more complete profile of HJV peptides in the extracts, the samples were subjected to PI-PLC treatment, which detaches the GPI-anchoring membrane from the protein-GPI complex and would make HJV peptides more exposed, and subsequently to immunoaffinity purification. Because of cross-linking of the bound anti-RGMc IgG to protein G on the surface of the beads by BS^3 ^the exposure of the cross-linked antibody may not be uniform. Also HJV exist in different forms. Therefore, capture of HJV peptides by the immunoaffinity beads may not be quantitative.

In liver samples (Figure 1B, 1-4), a thick band around 50 kDa as well as a faint band around 35 kDa were detected under non-reducing conditions, indicating the presence of 35 kDa HJV peptides which are not the large fragment of the heterodimer. Under reducing conditions a single band at 50 kDa and multiple bands around 35 kDa, one of which had a more intense signal than the others, were detected. The 20 kDa band was barely detected. In muscle samples (Figure 1B, 5-8), thick multiple bands around 50 kDa and also a very faint 35 kDa band were detected under non-reducing conditions. Unlike the case of liver samples, multiple bands around 50-55 kDa were detected but as with liver, multiple bands around 35 kDa, were detected under reducing conditions. The smudge was not detected. The multiple bands detected around 35 kDa under reducing condition indicate that the large fragment of the heterodimer in both samples may have microheterogeneity.

### Glycosidase Treatments

The revelation of a difference in the apparent molecular mass of HJV peptides in liver and muscle and the detection of possible microheterogeneity led us to study glycosylation in HJV. After removal of the membrane by PI-PLC and denaturation for glycosidase treatment, 100 C for 10 minutes in buffer containing detergent and reducing agent, the epitopes in HJV peptides appeared to be more accessible to the antibody; thus the positive signals became more intense, except for the 20 kDa peptide, whose positive signal became less intense after all treatments. In an attempt to detect minor positive signals, 60 μg of protein from each sample was analyzed by SDS-PAGE under reducing conditions. Mouse HJV consists of 420 amino acids (aa.): a signal peptide (aa. 1-32), a mature peptide (aa. 33-393), and a propeptide (aa. 394-420). There are three putative N-glycosylation sites at aa. 111, 206, and 365 in mature HJV. The auto-cleavage site which generates heterodimer is at 165-166. Therefore, the small fragment (aa. 33-165) may have one oligosaccharide branch and the large fragment (aa. 166-393), two branches.

### Peptide N-Glycosidase F

**Figure 2:**
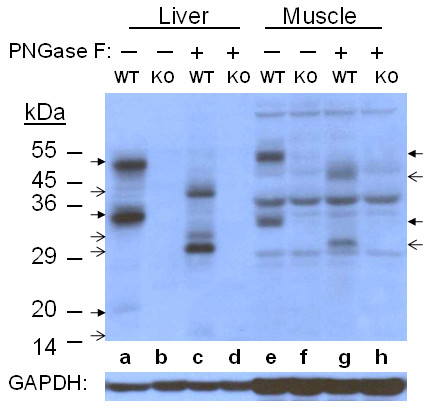
**PNGase F treatment of Liver and Muscle HJV**. PNGase F-treated and control samples containing 60 μg of protein were analyzed by 10% SDS-PAGE under reducing condition. HJV peptides were detected by immunoblotting. Thick arrows indicate control HJV peptides and thin arrows indicate digested HJV peptides.

PNGase F is an N-glycosidase that removes sugar moiety from asparagine residues at the attachment site of glycoproteins. The treatment provides the approximate molecular mass of naked peptides. In liver samples (Figure 2, a-d), a 50 kDa band, a thick 35 kDa band with a faint band just above, and a very faint 20 kDa band were detected in WT control. After the treatment the 50 kDa monomer band was reduced to a band around 42 kDa, and the multiple bands around 35 kDa were digested to a thin 32 kDa and a thick 30 kDa band. The thin 32 kDa peptide may be a final digestion product, not an intermediate to the 30 kDa peptide, since the two bands appeared in the same manner after treatments with a two-fold increase of enzyme concentration and/or reaction time (data not shown). The 20 kDa band was reduced to a band of 16 kDa. In muscle samples (Figure 2, e-h), non-specific signals were always detected in both WT and KO samples; however, the multiple bands of the monomer and the large fragment were clearly detected as thick 55 and 34 kDa bands only in WT control. The 55 kDa band was reduced to around 48 kDa and the 34 kDa band to 31 kDa, clearly demonstrating a difference in size of the deglycosylation products between liver and muscle samples.

### Endoglycosidase H

**Figure 3:**
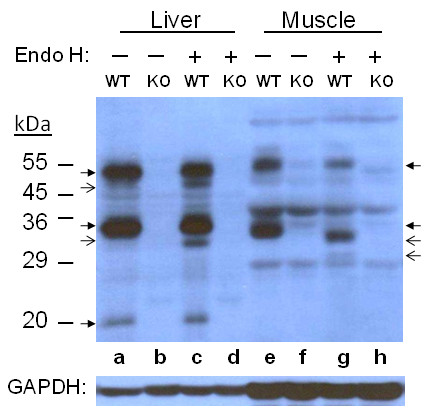
**Endo H treatment of Liver and Muscle HJV**. Endo H-treated and control samples containing 60 μg of proteins were analyzed by 10% SDS-PAGE under reducing condition. HJV peptides were detected by immunoblotting. Arrows are as in Figure 2.

Endo H cleaves mannose and oligosaccharides from N-linked glycoproteins. This treatment provides information on modification of the sugar moiety in the Golgi complex. In liver samples (Figure 3, a-d), after treatment a thin band appeared under both 50 and 35 kDa bands, at around 48 and 33 kDa, respectively, but the majority of both peptides appeared unchanged, as did the 20 kDa band. In muscle samples (Figure 3, e-h), the 55 kDa band became less intense but its size seemed unchanged, while the 34 kDa band was reduced to a thick 33 kDa and a very thin 31 kDa band. The profile did not change for treatment with a two-fold increase of enzyme concentration and/or reaction time (data not shown).

### O-Glycosidase and Neuraminidase

**Figure 4:**
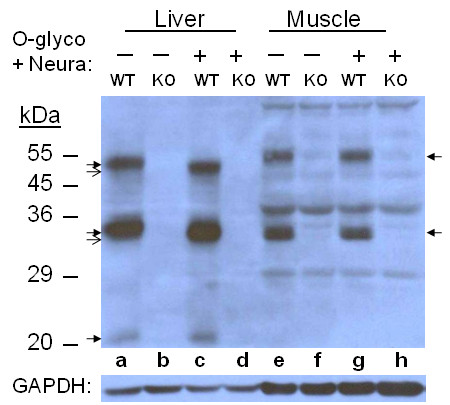
**O-Glycosidase/Neuraminidase treatment of Liver and Muscle HJV**. Glycosidase-treated and control samples containing 60 μg of preotein were analyzed by 10% SDS-PAGE under reducing condition. HJV peptides were detected by immunoblotting. Arrows are as in Figure 2

**Figure 5:**
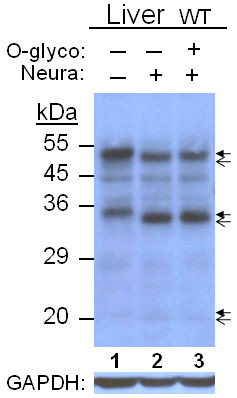
**Neuraminidase digestion of Liver HJV**. Aliquots containing 60 μg of protein from control (1), treated only with neuraminidase (2), and treated with neuraminidase and O-glycosidase (3) samples were analyzed by 10% SDS-PAGE under reducing condition. HJV peptides were detected by immunoblotting. Arrows are as in Figure 2

To check whether HJV underwent another modification besides N-glycosylation, O-glycosylation was tested by O-glycosidase which removes O-linked disaccharides from serine and threonine residues at the attachment site. Neuraminidase (sialidase) is an exoglycosidase that cleaves N-acetyl neuraminic acid residues (sialic acid) from glycoproteins. The former needs trimming of glycoproteins by the latter for its enzymatic action. In liver samples, a small size reduction of 1-2 kDa was detected for the 50 and 35 kDa bands but not clearly so for the 20 kDa band, while in muscle samples, no change was observed (Figure 4). Control digestion by only neuraminidase produced the same result for the two bands but the 20 kDa band also seemed digested, (Figure 5). Thus the size reduction was due to neuraminidase action, not to O-glycosidase.

## Discussion

Many glycoproteins are positioned at the cell surface and in the extracellular compartment, where cell-cell communication is occurring among various cell types in intact organisms [23]; HJV is one of them. Therefore, it is appropriate to use tissue samples to study the glycosylation of HJV. Our study using mouse tissue samples revealed complex glycosylation profiles of HJV, confirming both microheterogeneity and differential glycosylation in liver and muscle.

Under non-reducing conditions a liver HJV band was clearly detected but a muscle HJV band was barely visible, which indicates that muscle HJV peptides in the extracts are less exposed to the antibody than their liver counterparts. The 50-55 kDa monomer peptides of both liver and muscle HJV were still not clearly detected even under reducing conditions. Since HJV is a GPI-anchored protein, exposure of its epitopes may be influenced by interaction among HJV peptides, GPI, and the membrane [28]. It is likely that the surrounding environments, including GPI-anchoring, of HJV in liver and muscle are different. Under reducing conditions the 34 kDa muscle HJV peptide, a possible large fragment of heterodimer, was detected, but unlike what was found in liver samples, the small fragment was not detected. Instead, the 14-18 kDa smudge was detected under the both conditions. It might be that after auto-cleavage the muscle small fragment was processed in such a way that it could no longer form disulfide bonds and/or be degraded into smaller peptides. It should be noted that the positive signal of the liver 20 kDa small fragment became less intense after treatments with PI-PLC and glycosidases. Previous studies also showed its weak detection [7]. Thus, the small fragment of the heterodimer may be unstable for detection by immunoblotting. After PI-PLC treatment detached membranes were removed by centrifugation. A much larger pellet was spun down from the liver than from the muscle samples, clearly indicating a difference in the membrane structure for GPI anchoring between the tissues. When the extracts were ultracentrifuged, 80,000 × g for 45 minutes, HJV bands were detected only in the pellet from which the HJV peptides were released by subsequent PI-PLC treatment [29]. Therefore, the positive signals are of membrane-anchored HJV. It is possible that concentrations of soluble HJV in the extracts were too low to be detected by the immunoblotting employed for this study.

Profiles of immunoaffinity purification (Figure 1B) revealed not only the size differences of HJV peptides in the two tissues, but also differences in peptide form, indicating microheterogeneity of attached oligosaccharides. Interestingly, only the liver monomer appeared differently as a single band or possible multiple bands of very similar masses. Detection of the peptides around 35 kDa in both samples under non-reducing condition, whose intensity was stronger in liver than muscle, clearly indicates the presence of intracellular intermediates and/or soluble HJV in the extracts. The presence of intermediate HJV peptides has been previously suggested [5]. Soluble HJV have been detected in serum and blood on human and mouse (40 and 50 kDa peptides) by immunoaffinity purification [8] and in human serum (33 and 43 kDa peptides) by immunoprecipitation [21]. The apparent size difference of 50-55 kDa band between the reducing and the non-reducing samples may be due to disulfide bonding in the monomer under non-reducing conditions which makes the peptide more compact and lets it move faster in the gel. A similar observation has been reported elsewhere [30]. It is puzzling that although immunoaffinity purification indicates that 34 kDa muscle large fragments are in heterodimer form under non-reducing conditions (Figure 1B, 5-8), corresponding small fragments were not detected under reducing conditions (Figure 1A, 7).

The heterodimer is an auto-cleavage product from a mature protein [14], thus both the monomer and the heterodimer possess three N-glycosylation sites. But they are differently processed as their final forms on the cell surface are different. Therefore, it is possible that mature HJV peptides which eventually convert into heterodimers are differently glycosylated from those that remain monomers. The majority of the muscle monomer was reduced to around 48 kDa and the liver counterpart to around 42 kDa by PNGase F treatment, which also suggests the presence of other modifications, including other types of N- or O- glycosylation, and/or processing, *e.g*. splicing, that produce muscle HJV with a higher apparent molecular mass. The mass difference may also be due to variation in the composition and size of the GPI anchor. It is clear that the large fragment of the heterodimer has different oligosaccharide branches in liver and muscle since the 35 kDa peptide of liver HJV was reduced to 30 kDa, while its 34 kDa muscle counterpart was reduced to 31 kDa. PNGase F treatment removes nearly all types of N-linked oligosaccharides from glycoproteins; it therefore provides approximate molecular mass of proteins based on amino acid residues. The theoretical molecular mass of the large fragment is approximately 25 kDa (aa. 166-393), which also indicates possible involvement of other modifications. The 20 kDa liver peptide was reduced to around 16 kDa, which corresponds well with a theoretical molecular mass of 14 kDa for the small fragment (aa. 33-165).

Glycoproteins that undergo modifications in the Golgi complex may become Endo H-resistant. Therefore, the appearance of a slightly smaller band under both the monomer and the large fragment in the liver samples suggests that there is a small fraction of Endo H-sensitive HJV whereas the majority is Endo H-resistant. On the other hand, all of the muscle large fragments were digested, and thus are Endo H-sensitive, which is explicitly different from what was seen for liver. The detection of the very faint 31 kDa band in muscle samples indicates that a small fraction of the fragment undergoes very different modification from the rest. It is clear that microheterogeneity exists in the large fragment in which the second and third N-glycosylation sites are located in both tissues. Any small size reduction of the 55 kDa muscle monomer may not be clearly detected due to the concentration of acrylamide (10%) in the gel, which is suitable for separation of 20-40 kDa peptides. The finding that the liver HJV is neuraminidase-sensitive while its muscle counterpart is resistant also points to differential modification in the Golgi complex in the two tissues. Different size reductions by deglycosylation have been reported for cultured cells, depending on cell type [7,13,30]. The level of mRNA of RGMc/HJV in skeletal muscle in mouse was found to be much higher than that in liver [19]. However, detection of HJV by immunoblotting for liver and muscle samples in our study appeared comparable, thus HJV level in the total protein composition in both tissues is similar, indicating a difference in post-transcriptional control between the two tissues. A recent report that liver hepcidin expression is not affected by loss of HJV in skeletal muscle [31] indicates different roles of HJV from liver and muscle, respectively, which might well be designated by differential glycosylation.

It should be noted that although the first and third N-glycosylation sites (aa. 111 and 365, respectively) perfectly align among the mouse RGM family, the second site of HJV/RGMc (aa. 206) does not align with that of RGMa in the conservative von Willebrand factor type D domain (aa. 154-216), and RGMb does not have a N-glycosylation site in the central region. To our knowledge, the unique second N-glycosylation site and the proprotein convertase cleavage site set HJV/RGMc apart from the other RGM members. According to a structural model of RGM protein [32], the second N-glycosylation site lies near the central core and the third site is located at the far end of the structure. Therefore, it is possible that the sugar moiety attached to the second site influences the conformation of the peptide and its interaction with molecules in the vicinity. It has been reported that the induction of hepcidin expression only requires a small amount of HJV [33]. Therefore, it is tempting to speculate that only a small fraction of HJV with distinct oligosaccharide branches participates in the signaling pathway of hepcidin expression.

## Conclusions

HJV peptides of both liver and muscle were detected as multiple bands and glycosidase treatments confirmed microheterogeneity of HJV in both tissues. In addition, the large fragment of the heterodimer was found to have different oligosaccharide branches in liver vs. muscle. Therefore, since it is known that the second N-glycosylation site in the large fragment is unique to RMGc/HJV among the RMG family, glycosylation at the second site may be a key to elucidating distinct functions of RGMc/HJV in the two tissues, as well as in the RGM family more broadly.

## Abbreviations

GPI: Glycosylphosphatidylinositol; PI-PLC: phosphatidylinositol-specific phospholipase C; SDS-PAGE: sodium dodecyl sulfate polyacrylamide gel electrophoresis; Tris-HCl: Tris (hydroxymethyl) aminomethane hydrochloride; TBS: Tris-buffered saline; EDTA: ethylenediaminetetraacetic acid; DTT: dithiothreitol; PVDF: polyvinylidene difluoride; GAPDH: glyceraldehydes-3-phosphatate dehydrogenase; PNGase F: peptide N-glycosydase F; Endo H: endoglycosidase H; O-glycosidase: endo-a-N-acetylgalactosaminidase; BMP: bone morphogenetic protein.

## Authors' contributions

YF designed the study, performed experiments, and drafted the manuscript. JK helped prepare experiments and helped draft the manuscript. EN supervised the study and helped draft the manuscript. All authors read and approved the final manuscript.
